# From Research into Practice: Converting Epidemiological Data into Relevant Information for Planning of Regional Health Services for Refugees in Germany

**DOI:** 10.3390/ijerph19138049

**Published:** 2022-06-30

**Authors:** Maren Hintermeier, Andreas W. Gold, Stella Erdmann, Clara Perplies, Kayvan Bozorgmehr, Louise Biddle

**Affiliations:** 1 Section for Health Equity Studies & Migration, Department of General Practice and Health Services Research, Heidelberg University Hospital, 69120 Heidelberg, Germany; andreas.gold@med.uni-heidelberg.de (A.W.G.); clara.perplies@med.uni-heidelberg.de (C.P.); kayvan.bozorgmehr@uni-bielefeld.de (K.B.); 2 Department of Population Medicine and Health Services Research, School of Public Health, Bielefeld University, 33501 Bielefeld, Germany; louise.biddle@uni-bielefeld.de; 3 Institute of Medical Biometry, Heidelberg University Hospital, 69120 Heidelberg, Germany; erdmann@imbi.uni-heidelberg.de

**Keywords:** migration, healthcare planning, Germany, asylum seekers and refugees, research communication, health monitoring

## Abstract

Health data of refugees and asylum seekers (ASR) is not routinely collected in Germany. Based on health data of ASR collected in 2018 in regional accommodation centres, we developed a dashboard to estimate regional burden of disease in Baden-Wuerttemberg, Germany. We aimed to find out how scientific data can support actors involved in healthcare planning for ASR in Germany and, within this scope, to explore how healthcare planning is conducted in this context. We conducted 12 qualitative semi-structured interviews including a usability test for a health data dashboard with regional decision-makers. Results showed that healthcare planning processes for ASR in Germany involve a complex set of actors in both long- and short-term decision-making. Data gained from representative surveys can support long-term decision-making and thus support the resilience of the health system, but it must balance the need for simple data presentation with transparent communication of potentially complex methods.

## 1. Introduction

By the end of 2020, there were approximately 6.8 million asylum seekers and refugees (ASR) in Europe, of which 1.5 million were hosted in Germany [1]. In the same year, 417,000 first-time asylum applications were submitted in Europe [2], including 102,600 that were received in Germany [1]. Due to the experiences refugees have before, during and after their flight, health needs of this population need to be identified and addressed adequately [3]. However, many countries in Europe still struggle with providing healthcare access and integrating ASR in healthcare systems [3,4,5]. Information about health needs of ASR to facilitate needs-based healthcare planning is particularly important in this process.

However, health data is not routinely and consistently collected for ASR in most European countries [6,7,8]. While much focus has been on the improvement of health records and registry-based data for refugee health, a review has suggested that survey data may be a valuable resource for gaining insight into refugee health and accessibility of healthcare services, but this resource has not yet been adequately exploited or integrated into national monitoring efforts [6].

In Germany, the RESPOND project (Improving regional health system responses to the challenges of migration through tailored interventions for asylum-seekers and refugees; see Box 1) set out to collect representative survey data on health and healthcare for refugees at a regional level [9,10]. This was intended to complement national surveys such as the German Health Interview and Examination Survey for Adults (DEGS), which includes migrants but does not allow for identification of refugees [11,12] and the IAB-SOEP-BAMF Panel (joint survey by the Institute for Employment Research (IAB), the German Socio-Economic Panel (SOEP) and the Federal Office for Migration and Refugees (BAMF)), which is specifically designed for refugees but only offers a very limited set of health indicators [13]. The RESPOND survey comprised a random sample of 1% of all ASR living in the state of Baden-Wuerttemberg. A variety of health and healthcare-related questionnaire items and validated instruments were used in the self-completed pen and paper questionnaire [9]. Results showed a high health burden among the refugee population, particularly for mental health, with concurrently high unmet needs for primary and specialist care [10].

The question arises as to how this survey data can be made widely accessible and usable for healthcare planners. This is an important issue not just in the realm of refugee health, but also for population health more broadly. For instance, the German public health institute (Robert Koch-Institute) has recently published a data dashboard, which presents a number of health indicators derived from national surveys and visualises these by gender, age and federal state [14]. The context of healthcare planning for ASR in Germany, however, makes the communication of data a particularly complex task. The provision of healthcare is determined by the type of accommodation in which refugees live; generally, the accommodation system of ASR is split into state-level reception centres (RC) for the first months after arrival and into regional accommodation centres (AC), where ASR have to stay until the asylum process is decided. Within RC, healthcare is the responsibility of the state and often organised at the RC-site itself [15,16]. ASR are then dispersed to AC at a regional level, which are administrated by either the county or the municipality depending on the states’ accommodation policy system [17]. ASR living in AC usually receive medical care within the regular German healthcare system, but may face language [16,18], financial [18,19], geographic [10] or structural barriers [20]. Many different actors are involved in the accommodation and healthcare planning processes, which include state governments, integration authorities, and local health departments at the municipal level, as well as private and non-profit organisations (NGOs). This means that the communication of health data in this context addresses a wide variety of audiences with different backgrounds, professional fields and experience in using data.

Against the backdrop of this complexity of actors and processes involved in healthcare planning, the primary research question was how scientific data is able to support the planning of regional refugee health services. More specifically, we aimed to test the usability and usefulness of a health information dashboard (RESPOND-INTENT) based on the data collected by the RESPOND survey with the potential target group. In order to judge how the dashboard can be used, we needed to explore how healthcare planning for ASR in Germany is conducted at a regional level (i.e., in regional AC) and what information is needed or used by the actors involved. We understand healthcare planning as the sum of all actions taken by different actors relating to the provision of health services for refugees, including health prevention, needs-based healthcare planning and the organisation of administrative tasks related to the financing of healthcare and other related tasks such as accommodating ASR with special health needs. We also take interactions between different actors in this process into account [21,22]. Therefore, the secondary research questions explored were: (i) what decisions are made regarding the planning of healthcare for ASR, (ii) how are these decisions made, (iii) what kind of information is required for decision-making, and (iv) how is the health dashboard RESPOND-INTENT rated concerning its usability and usefulness.

Box 1 **Data collection of the RESPOND survey.**

**Aims of the RESPOND survey:**

The RESPOND survey “aimed to collect valid epidemiological data on refugee health status and healthcare provision” [10]. The health monitoring survey was conducted in the state of Baden-Wuerttemberg, Germany.

**Data collection:**

Multilingual field teams collected data using a door-to-door approach between February and June 2018 in Baden-Wuerttemberg. Participants were eligible if they were 18 years or older and spoke one of the nine study languages (English, German, French, Farsi, Arabic, Turkish, Russian, Serbian, Albanian).

**Sampling strategy and response rate:**

A random sample of 65 out of 1938 accommodation centres with a net sample of 1% of all ASR living in the state was drawn to ensure a representative sample. Finally, 412 participants out of 58 AC were included. This equals a response rate of 41.7% for adults and a participation rate of 49.4%.

**Health variables:**

A variety of health and healthcare-related questionnaire items and validated instruments were used in the self-completed pen and paper questionnaire. The following variables were used for the development of the RESPOND-INTENT dashboard:

*Physical health:*

- European Health Interview Survey (EHIS), general health, pain, chronic illness

*Mental health:*

- Patient Health Questionnaire-2 (PHQ-2; depression) and General Anxiety Disorder-2 (GAD2)

*Cardiovascular disease:*

- Incidence of stroke, coronary heart disease, angina pectoris, heart failure, hypertension [9,10]

## 2. Materials and Methods

We conducted 12 semi-structured interviews (11 individual and one group interview) with actors involved in healthcare planning for ASR in regional AC from May to July 2021. Ethical approval for the study was obtained from the ethics committee of the Medical Faculty Heidelberg on 12.10.2017 (S-516/2017).

Prior to participant recruitment, a dashboard illustrating health burden of ASR living in Baden-Wuerttemberg was developed based on the data collected in the scope of the RESPOND survey (Box 2). The dashboard intends to give an overview of physical, mental and cardio-vascular health needs of ASR at a regional level. For this purpose, RESPOND survey data was fitted to county-level sociodemographic characteristics using iterative proportional fitting [23]. 

We chose a purposive sampling strategy to recruit participants [24]. Initial recruitment took place through the research network of the RESPOND project, which included an information session at a RESPOND project event and an invitation to participate circulated to all regional offices responsible for the accommodation of ASR in the state of Baden-Wuerttemberg. Subsequently, we used the snowballing method and asked interview partners for contacts of other eligible and interested persons. We aimed for a heterogeneous sample in geographical location and affiliations of actors (integration and migration offices, health departments, NGOs). Moreover, we included three participants from other federal states to assess similarities or differences in healthcare planning for ASR between the states.

Participants were informed verbally and in writing about the aims of the study and handling of their personal data. After written consent, interviews were recorded. The interview-guide was comprised of two parts (Supplementary File S1). The first part included questions about the actors’ position and fields of activity, the actors’ involvement in healthcare planning and possible challenges and decisions that needed to be made, and about data used and needed for planning and/or decision-making. In the second part of the interview, we conducted a formative usability test [25] to examine the usefulness and usability of the RESPOND-INTENT dashboard and the needs for further development using the think aloud method [26]. Interview partners were guided through the dashboard based on a simple scenario after sharing their first impression on the user interface.

Interviews were conducted in pairs (by LB, MH, AG, CP and a fifth female research assistant) via video or phone and lasted between 48 and 77 min. After a verbatim transcription, interviews were pseudonymised and analysed with MAXQDA 2020 [27] using a thematic analysis approach [28]. Initially, a code system along the topics raised by the interview guide (healthcare planning, decision-making, database used/needed) was generated deductively. This code system was applied to 12 interview transcripts in parallel by MH, AG and LB and subsequently refined to a more finely differentiated code system (Supplementary File S2) in a series of analysis meetings. The remainder of the interviews were subsequently coded by MH using this code system. Coded transcripts were analysed for key themes and emerging patterns in a series of discussions by MH, AG and LB. These are reported below following the structure of the secondary research questions for this analysis.

Box 2 **The RESPOND-INTENT dashboard.**

**Overview**

The RESPOND-INTENT dashboard shows health estimates for asylum seekers and refugees (ASR) living in accommodation centres in the state of Baden-Wuerttemberg, Germany. The data for this dashboard was estimated at regional-level using state-level results from the RESPOND survey. A map of Baden-Wuerttemberg builds the core of the dashboard. By choosing a health indicator (physical health, mental health or cardio-vascular diseases), counties in the map are shown in shades of red or green according to the deviation from the mean of the health indicator for all ASR in Baden-Wuerttemberg. On the left side, a legend explains the colour scale and on the right side, distribution of gender, age and region of origin for the chosen region is displayed. At the bottom right is a field to insert the distribution of ASR manually and calculate estimated health needs accordingly. Underneath the map are buttons to receive information on method, health indicators, data sources and regions of origin.

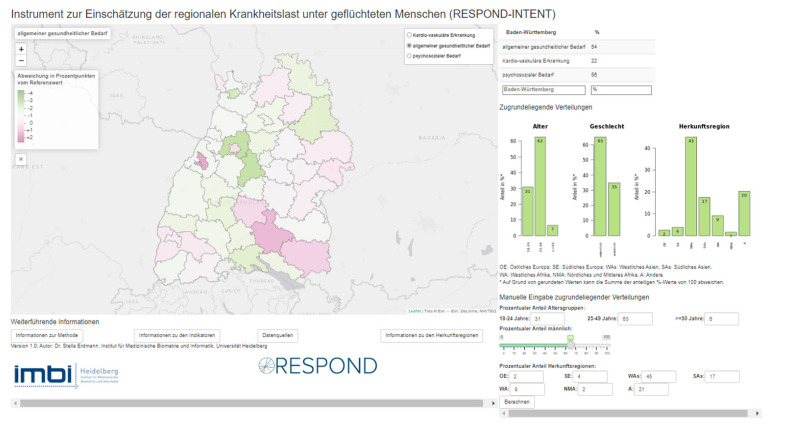

**Dashboard development:**

The dashboard was developed based on a raking Algorithm to show the estimated county average of health needs of ASR living in respective states according to the health variables listed in Box 1.

**Data sources:**

Besides the RESPOND-data, we used two more data sources namely:

- Data of counties on all ASR living in Baden-Wuerttemberg stratified for sex and nationality (© Statistisches Bundesamt (Destatis), 2020|state: 18 March 2020/08:33:28; reporting date: 31 December 2018)
- Data of age-groups of recipients of asylum seeker benefits from the regional data-base (© Statistische Ämter des Bundes und der Länder, Deutschland, 2020; reporting date: 31 December 2018)

Out of these sources we created a hypothetical database of 2018 (We chose 2018 as RESPOND survey data collection was in 2018.) including all adult ASR (>17) per county stratified for sex and nationality.

**Data preparation:**

In a weighting procedure (the so-called “iterative proportional fitting”), the distribution of the socio-demographic parameters of participants in the RESPOND survey was adjusted to the distribution in the districts. This means that the values given (=estimated prevalences) reflect the uneven distribution of sociodemographic characteristics of refugees at the district level—and the associated differences in health needs.

**Implementation in R:**

The data management, analysis and implementation of the dashboard was done in the statistical programming language R [29] (around 600 lines hard code) and used the following R packages: shiny [30] and shinyWidgets [31] (for building interactive web applications with R), leaflet [32], leaflet.extras [33] and tmap [34] (for creating thematic interactive web maps), haven [35], readxl [36] and sf [37] (for importing/reading/exporting files), RColorBrewer [38] (for using colours that are also easy to recognise for colour-blind people) and mipfp [39] (for application of the iterative proportional fitting approach) [40].

## 3. Results

### 3.1. Description of Sample

The 14 interview partners were from eight different counties in Baden-Wuerttemberg and three other federal states (Table 1). Informants worked in different organisations (office for integration and migration, health department, city administration or NGOs), and in different positions reaching from leadership positions as team managers to working directly with ASR, e.g., as a refugee health nurse. We operationalised these differences with the characteristics “operational” and “organisational” (Table 1, column 7). “Operational” level actors were involved in healthcare planning at an individual level and worked directly with ASR. Informants assigned to the “organisational” level were involved in healthcare planning in a managerial capacity. The professional background was diverse, including health-related studies, nursing, psychology, sociology, politics, management, pedagogy, social work and intercultural studies.

Informants described their involvement in healthcare planning in line with their fields of activity. For example, participants from offices for integration and migration were directly involved in the accommodation of refugees while participants from health offices had a broader remit and did not exclusively work with the refugee population group. At an operational level, tasks comprised identifying healthcare needs, handing out healthcare vouchers, coordinating physicians’ appointments or arrangement of cost coverage. Tasks described by participants working in an organisational or managerial capacity differed by organisational affiliation. Actors working in health departments listed initial examinations (as established in the German asylum law §62 AsylG) or infection protection including vaccination coverage. Infection protection was primarily planned when requested by the offices for integration and migration, e.g., during disease outbreaks. Participants working in offices for integration and migration primarily managed appropriate accommodation for newly arriving ASR, with one participant stating that health was rather a secondary concern:

*“The coordination with regard to the topic of health is only possible to a limited extent and we simply have to see where we have space for residents when we receive allocations from the initial reception centres of course, our first priority is the question of what kind of accommodation is best suited for residents, individuals or families with what needs, and only then, in second place, does the supply at the location also play a role.”*

(P3)

Moreover, some participants from offices for integration and migration reported tasks like initiating projects for needs assessments or establishing new accommodation facilities, while others were involved in committee work or public relations activities to achieve structural changes. At the same time as describing such activities, some interview partners emphasised that healthcare planning—understood as needs-based planning—was neither in their area of responsibility nor in the health offices’ remit:

*“In my area of responsibility, there is no planning of health… the healthcare… of people with a migrant background. I am also not aware of any systematic planning on the part of the health department or other offices.”*

(P7)

### 3.2. Types of Decisions Made

Actors described a variety of different planning decisions, but frequently did not explicitly frame these as “healthcare planning”. Decisions could be distinguished as being either short-term or long-term. Both actors with organisational and operational tasks mentioned short-term decisions, whereas long-term decisions were only reported if actors were involved in organisational tasks. 

Short-term decisions were characterised by their immediate response to a practical problem in the provision of health and accommodation services. In response to an under-provision of specialised mental health services, for example, one participant (P7) reported building a cooperation with a psychosocial centre specialised in the care for refugees to address pressing mental health challenges:

*“If any needs have been reported so far, then it was immediately tried to involve any specialised institutions or to start some kind of cooperation, simply in order to be able to cover the needs. Yes, like it was with the social psychiatric services, for example. The need was acute and the corresponding [psychosocial centre] was called in and the cooperation was organised. We were able to cover some needs well in this way for others there were no capacities left.”*

(P7)

Other short-term decisions included the development of health communication training for staff during the COVID-19 pandemic, decisions on granting special treatment like rehabilitation, expensive therapies or medical aids, and the provision of adequate accommodation for refugees with special health needs. For example, one informant (P4) reported that they had to decide within two weeks if they would build a ramp to accommodate a family with three children in wheelchairs taking the risk to bear the costs themselves as municipality.

*“We have to accommodate the people accordingly and then, in the same breath, so to speak, we are informed or have been informed that three of the five children are in wheelchairs. And then we have to accommodate them. (…) We were actually lucky that we had a suitable ground floor flat that we could make available to the family. We then had a ramp built, also at our own expense, to create access via the balcony, because there were two steps to climb.”*

(P4)

Besides the timely manner of short-term decisions, another quality was that our participants either could decide directly or were directly involved in the decision-making process. In contrast, long-term decisions required agreement of higher-level authorities and were often decided accordingly at a higher hierarchical level. The majority of long-term decisions for health concerned decisions on a thematic focus for health projects as well as the financing of services or personnel.

*“But as soon as it came to psychological problems, we realised that there was almost no access to the regular systems. (…) And we said that this must change urgently, because the number of people with psychological problems or disorders has simply increased in the last two years. (…) And we have responded to this with this personnel recruitment.”*

(P5)

Adding to this, P1 described the long-term decisions made in their organisation and distinguished between healthcare planning-related decisions (e.g., financing of services) and decisions on accommodation processes.

*“In the area of health, I don’t even see the big decisions. For example, I think it is important that we support a project for traumatised refugees in the area of health (…). Because we saw that otherwise we wouldn’t be able to (…) treat people who otherwise couldn’t or wouldn’t want to go to treatment. These are the things that can be decided on a smaller scale. The difficult overall decisions are: what is the prognosis, how many refugees are coming, how many are leaving, how much accommodation capacity do I need, and if I no longer need a capacity, dismantling the accommodation and thus also dismantling staff, not only in social services, but in principle. So, just as we built up in 2015, we will also reduce again. These are the decisions.”*

(P1)

Other long-term planning decisions included the improvement of accommodation facilities to prevent illness and promote health. The reduction of occupancy of reception centres during the COVID-19 Pandemic was one example:

*“For example, corona (…) has indeed influenced our lives very much and also made them very difficult. But at the same time, many have also benefited from it. One example is, with a lot of discussion, we managed to reduce the number of refugees in a reception centre. (…) And that was also done politically, had to be agreed, [among the different parties]. And we had a lot of discussions about that.”*

(P14)

Participants stressed the importance of cooperation between all actors in the long-term decision-making process. Due to the heterogeneity of actors involved in decision-making, including county-level politicians, planning processes involved an alignment of priorities between individuals and offices with potentially diverging mandates:

*“What effect do we want to achieve together, in the district or in our community, what do we want to work towards together?”*

(P10)

Crucially, long-term planning required decisions on how to spend available budgets, which were often not in the hands of the interview partners. Instead, individuals tasked with the provision of care for ASR had to advocate for their cause at a county-level:

*“The municipalities in our district pay a district levy. And the goal is to keep the district levy (…) in a fair ratio, so that the municipality can also, I don’t know, build a kindergarten. Yes, that’s the dilemma the district faces, we can’t just decide for ourselves.”*

(P1)

### 3.3. Decision-Making Processes

The processes by which decisions were made also differed between short-term and long-term planning. Regarding short-term planning, decision-making was often based on locally reported needs, such as issues raised by personnel or officially reported incidents:

*“You notice [a health issue] by how many police incidents you have, by how many disturbances within the accommodation you have, by how often a social worker says: ‘I have someone here, I can’t reach him at all with normal speech and writing, because he’s off somewhere in his head’.”*

(P5)

In this case, actors translated the issues raised into a need for mental health professionals within the accommodation facilities. Therefore, the team of our informant collected data (*“we made a list”* (P5)) to argue the case towards the county government. 

In the example of the ramp construction (see Section 3.2), cooperation between the local authority real estate office and the district administration took place to find needs-based solutions. Other actors reported collaboration with different service providers to meet residents’ healthcare needs. In these instances, short-term decision-making required good networks and collaboration in order to solve problems quickly. 

In contrast, long-term decisions were made differently. Decision-making processes for these kinds of decisions took longer and required negotiation work. Negotiation took place with political partners, in committees, in the scope of in-house meetings or directly with the head of the administrative authority.

*“Then I report it to my superior, who then forwards it to the head of the office, and then we see what conditions [are given] and to what extent we can change it, whether it is somehow offered by the ministry [government] or whether there are any offers here in the district that we don’t know about yet, or whether we have to do something to change it. So sometimes, it is tied to political decisions/sometimes there are infrastructures and sometimes there is nothing.”*

(P12)

However, informants stated that there is little to no systematic data on health of ASR.

*“(…) there is a lack of scientific data and study basis. Especially for the health of refugees. And health data that is also collected explicitly for the group.”*

(P10)

As a result, actors drew on a variety of information sources to produce convincible arguments. For example, actors that advocated for the financing of personnel or new projects described using their own project documentation or aggregated documentation from social workers. In most cases, the eventual decision-making authority was usually not with our informants themselves but with higher-level authorities like political committees, budget holders or the administrative head.

*“(…) in the end the politicians, the local politicians, the local council is the one who has to say, has to decide on the basis of the data we give them, from the health reporting, from the documentation we do on the project.”*

(P13)

Across short and long-term decisions, participants reported good networks and cooperation between different actors as being key to quick decision-making. Important collaboration partners included psychosocial centres, health departments and NGOs, but also the police and the local authority property office. However, several participants mentioned a lack of cooperation between partners and, in some instances, received essential information only upon request. This resulted into a wish for improvement in networking and exchange:

*“Because what I think is still a major shortcoming in health promotion for refugees or immigrants in general is that too little… exchange happens in a structured way. It is selective, like you know people. But I believe that this intersectionality should be expanded more strongly.”*

(P2)

Rigid data protection requirements and unavailable skills and resources for data analysis were perceived by several participants to be further hindrances to accessing and using available data for appropriate (healthcare) planning.

*“So, data protection, yes, unspeakable sometimes in this field… because actually it also prevents, it’s also a certain hindrance.”*

(P1)

### 3.4. Data Required for Decision-Making

While all participants reported using some information or data sources in their decision-making processes, many participants reported large information gaps and expressed the need for more comprehensive, timely and rigorous data sources.

Given the quickly changing context of accommodating and planning healthcare for ASR, especially those actors reporting short-term planning expressed a need for improved data transfer from RC. Apart from the data transfer from RC, participants emphasised that more specific health information on newly arriving ASR, including potential infectious diseases, mental health concerns, healthcare needs or other special needs should be communicated.

*“It would of course be nice for us the data transfer would be a bit more intensive then we would know exactly what kind of people we would have to adjust to and what we would actually have to offer.”*

(P3)

Moreover, the importance of the timeliness of this data was raised. P4 for example reported that health needs were communicated only two weeks in advance, which made the provision of adequate accommodation a challenge. 

Participants who were involved in long-term decision-making reported the need for reliable data used to identify priorities and make the case for increased service provision or recruitment of additional personnel to budget holders.

*“In Germany we have a great lack of translation of health data. So that one can, so to speak, gain the health data, because many people experience a lot or know a lot, but (…) you cannot use that. (…) So the question is always how well you can link [experience and knowledge] to really get real data, to find out about certain problem areas.”*

(P14)

Systematically collected data on health and healthcare needs of ASR were needed for this process but not currently available. Because data often need to be communicated to actors and politicians who do not necessarily have a health background, the presentation of information needs to be simple and clear:

*“To be quite honest, the bare figures were helpful because everyone understands them. And we have to be aware that in our committees, in the district council or in the municipal committees, there are people who have been elected. They may not have any in-depth knowledge of this topic. This means that it has to be presented in a relatively simple way.”*

(P1)

P1 addressed the global political climate as relevant information to estimate what the access numbers of ASR for the coming months might look like and thus be able to manage human and other resources needed for adequate accommodation of ASR. Other participants further stressed that data needs to be particularly reliable and robust in the context of refugee health because of the political sensitivity of the topic. Data could easily be misunderstood or even misconstrued if it is not reliable and robust enough to make clear statements:

*“And especially when there are political forces that are rather against refugees and do not want to invest money there. I think it is then also a higher responsibility, […] to make very transparent and easy to understand what [data tools] can and cannot do.”*

(P2)

Finally, informants indicated that it would be helpful to have a common information platform where all relevant data for decision-making can be pooled.

### 3.5. Usability and Usefulness of RESPOND-INTENT Dashboard

A summary of the technical and content-related requirements for data dashboards expressed by participants is provided in Table 2. It also summarises the practical implications of these requirements and assesses whether requirements have been met by the RESPOND-INTENT dashboard.

#### 3.5.1. Usability

Initially, all participants needed a few minutes to get familiar with the dashboard but were able to describe and develop a basic understanding for the functions and content shown. The three indicators: general health needs, mental health needs and cardio-vascular diseases were perceived as being useful, although the latter was not always a familiar term. For some users, the compact presentation of information (including title, colouring, figures or functions) given by the dashboard was somewhat overwhelming and they would have liked an introductory text or brief description (cf. Table 2). However, a dashboard, which is easy and intuitive to understand, was highlighted as being particularly important given the scarcity of time in everyday work:

*“Because people from the field often don’t have much time, they want information quickly.”*

(P10)

All participants welcomed the idea of the dashboard, but not all users intuitively understood it. In this respect, a common query was the need for a comparative value to the percentage values given for each county, such as the corresponding figure for the general population (cf. Table 2). More than half of the participants asked for background information or a guide to use the dashboard and did not intuitively notice the information provided in the bottom bar of the window (see Box 2 or Supplementary File S3). Interviewees stressed the importance of understanding the background and rationale of the data displayed.

*“And I know that in the past, when we didn’t work carefully, we suddenly argued with things that simply weren’t true and that’s why you always have to be clear when you work with data: Ok, are they all correct, can I read them well? And if that is not the case (…) then you can just quickly draw conclusions that are not at all reliable in reality. I consider that difficult.”*

(P5)

At the same time, there was a strong wish for a simple and clear display of the analysed data. 

Desired characteristics for a simple data presentation were the use of comprehensible language that even a layperson can understand and using graphics or tables for display.

*“Yes, so in three parts it is best. First of all to have tables or graphics, then someone introduces me to what I see on these tables and graphics and at the end there is a summarised sheet with ‘this is why you should do this’.”*

(P7)

P7 additionally suggested recommendations for actions as a useful form of data presentation for the decision-making process.

#### 3.5.2. Usefulness in Everyday Work

Regarding the usefulness of RESPOND-INTENT in the everyday work, meaning the possible usefulness of the dashboard in the actors’ fields of activity, we must distinguish between users involved in short-term and users involved in long-term planning. Participants involved in short-term planning, who were often working directly with ASR, did not see a considerable use of the dashboard in their everyday work. Several participants stated that the district data displayed in the dashboard is not detailed enough to support individual healthcare planning.

*“So, it’s just very broad and generic. (…) That’s why I have to be honest and say that we look more at the individual case (…) what does [the person] have and what does [the person] need? And from that we conclude: oh, we have a larger group and for the larger group we have to do something.”*

(P1)

However, they saw the relevancy of the dashboard as being a source of background information, a source for discussions or projects and for their own interest.

*“I think it makes sense, and is also necessary, in order to get a very good, general overview at first. We don’t have any detailed data here, but we can already derive certain trends, so to speak.”*

(P10)

For participants involved in long-term planning the dashboard served as a useful overview of a new or relevant topic, e.g., for internal or external discussions or network meetings. In addition, others could imagine that it would be a useful source of information when drafting project proposals, making the case for a re-prioritisation of allocated funds or advocating for additional resources.

*“I would use it as a basis in my work and (…) maybe I would also use it in meetings when it comes to financial topics, because I always need a good basis and a good line of argumentation and thus create a basis [for that]. So that would already be something where I think one could work with it and also take up arguments…”*

(P3)

To improve the dashboard and its usefulness in everyday work, participants suggested some further additions and extensions (cf. Table 2). In particular, users suggested a function for comparing prevalences among ASR to those of the general population as well as other vulnerable groups. Participants further suggested expanding the dashboard to cover children’s data and more specific health outcomes, such as depressions or anxiety rather than general mental health. Due to the rapid changes in numbers and sociodemographic composition of ASR arriving at the regional level, the importance of using recent data sources was also raised; our data from 2018 was considered as being outdated by some. 

Overall, several participants had questions related to the data collection context and type of data (e.g., survey vs. health insurance data). They noticed that the dashboard only refers to health needs of ASR living in AC and thus emphasised the difference between health needs of people living in AC and those in subsequent accommodation, and the need to distinguish between these groups of people for the purposes of healthcare planning:

*“The situation at the beginning is of course completely different and the need is also different than if, for example, people with a migrant background who have been in Germany for a very long time were to be included or who are perhaps already in subsequent accommodation for which integration managers are responsible.”*

(P9)

## 4. Discussion

The results of this interview study provided insights into the way in which actors involved in short- and long-term healthcare planning for ASR used data sources and whether health data presented in the form of a data dashboard was useful in the planning process. 

Actors in short-term planning were mainly entrusted with operational tasks and worked for integration and migration offices, municipalities or NGOs. Data requirements for short-term planning were specific to the ASR in their care, such as age, sex, nationality, health status and special needs. Healthcare planning took place at an individual level and the decision-making power was with our informants. Regional health data on ASR as provided by our dashboard was not considered useful for short-term planning, but did provide actors with background knowledge of the broader health trends for this population. Actors involved in long-term planning mainly worked for integration and migration offices or municipal health authorities and worked particularly at an organisational level. Long-term planning required data to build arguments towards higher-level leadership, as long-term decisions were made by committees, head of the administration or budget holders. Participants perceived health data on ASR as displayed in our dashboard as useful for this purpose provided that the information is based on sound data analysis and the results are communicated in a simple and comprehensive way.

However, the dashboard was not always simple and comprehensible for all participants. Due to the high heterogeneity of actors involved in the health context of ASR, not all have the required health background to accurately interpret the figures shown by the dashboard. For example, several actors struggled with the concept of prevalences or with medical terms such as “cardio-vascular disease”, demonstrating that health dashboards need to be rigorously user-tested and accompanied by simple and clear explanatory statements. Our dashboard used extrapolated survey data to give an impression of the health trends for ASR living in the region using representative data. However, several participants interpreted the given figures as originating from health insurance records and thus came to unintended conclusions about what the dashboard showed. There was a very limited understanding of the type of data which was (i.e., survey data) and was not (i.e., health insurance data) available in the field of ASR health, and thus what data sources could reasonably be used for the purposes of planning. When developing health information dashboards, developers need to take not only the intended use but also the data literacy or knowledge boundaries [41] of the target population into account in order to avoid false conclusions being made. Overall, participants expressed the need for the data presentation to be as simple as possible.

At the same time, however, participants also stressed the need for data sources and methods to be rigorous. While this is pertinent for all health communication, participants stressed the particular importance in the context of ASR health due to the political sensitivity of the topic. Data must be generated in a reliable way and communicated transparently so that methods can be understood. Furthermore, participants stressed the need for data comparisons, including comparisons among the group of ASR (e.g., by age, nationality, educational level) as well as with other population groups. These aspects are required so that results may not be misconstrued and used to shore up anti-migrant sentiment in political debates. Squaring the requirement for transparent communication and rigorous methods with the requirement for simple reporting, however, is no easy task, especially in the realm of survey methodologies, which often include complex sampling and weighting procedures. 

Previous research from the field of evidence translation shows that scientifically gained evidence cannot simply be “presented” to decision makers, but that studies should be designed with the use of the results by decision-makers in mind and have ongoing consultation throughout the research process [42]. There are different frameworks and models presented in the literature that may support or guide the implementation of knowledge translation in research [41,43,44]. Knowledge translation has proved to be a key element in this decision-making context, where a “one-size-fits-all” approach did not appeal to everyone. Instead, we found that the proposed data dashboard served the interest of a specific audience of decision-makers with very specific requirements. Going forward, the development of such tools should be carried out in tandem with decision-makers. This process should include a discussion of how to balance the need for rigor of methodology and simplicity in the presentation.

Our research demonstrated different information needs for those actors involved in short- and long-term planning, with the former finding the information provided by our data dashboard less useful. Arguably, however, several of the issues, which require short-term decision-making, could be avoided if authorities were able to take a more long-term perspective on health matters, including, for example, more adequate provision of mental health services to match the high need among ASR. Notably, many of the participants interviewed for this study did not understand their professional activities as healthcare planning, even though the activities described had clear implications for the healthcare of ASR in their responsibility. In this sense, improved health monitoring with rigorously collected survey data could contribute to the resilience of the health system for ASR by improving the “capacity to combine and integrate different forms of knowledge” [45] and make it easier for authorities to base their long-term decision-making on a sure evidence-based footing. At the same time, however, it is unlikely to resolve the need for all short-term planning. As such, data from monitoring surveys must necessarily be combined with other forms of knowledge, including timely and reliable information on new arrivals for the provision of ground-level services [46], but also through improved accessibility of medical records (e.g., RefCare Manager [46]) and health insurance data [7,14].

This study benefitted from researchers who had substantial prior research experience in ASR health services in Germany and were thus able to understand the complexities of the context. The heterogenous sample enabled a broad insight into the decisions made concerning healthcare planning for ASR. Our study is limited by the small sample of 14 participants (12 interviews) some with little professional experience in the field (1–3 years). However, through the connection to the research network of the RESPOND project, it was possible to recruit a heterogeneous sample of relevant and representative participants in this field. Unfortunately, we could only speak with three representatives of health departments due to the COVID-19 pandemic. Another limitation was the small number of participants recruited from other states, but we do not expect these to differ substantially given the commonality of themes raised in our sample. These limitations to our sample mean that the presented results on our secondary research question, namely how healthcare planning is conducted for ASR, are exploratory in nature. The integration of the usability test into the interviews provided valuable additional insights into the use of a data dashboard by our participants, but was limited by some technical issues in the video call format. Technical issues related to problems viewing the dashboard due to incompatibility of the dashboard with the browser used by the participants or in individual cases due to firewall issues, screen size or poor internet performance. In these cases, the interviewer had to share the screen, which limited the interaction of users exploring the dashboard as they could not use the mouse. Overall, however, we found video calls to be a feasible way of conducting qualitative interviews. 

## 5. Conclusions

Healthcare planning processes for ASR in Germany include a complex set of actors involved in both long- and short-term decision-making. Data gained from representative surveys can support long-term decision-making and thus support the resilience of the health system, but it must be presented in a simple way and based on rigorous evidence in order to be useful. The research process needs to consider policy makers interests’ and challenges, and provide clear, targeted and comprehensive data presentation, which balances the need for simple data presentation with a transparent communication of potentially complex methods. Further research should aim to comprehensively understand how healthcare planning for ASR in Germany is done and investigate the best ways of involving decision-makers in the research process, as well as finding new ways to make better use of other existing data sources, including medical records and health insurance data. 

## Figures and Tables

**Table 1:** Sociodemographic characteristics of participants.

Participant	Age Group	Gender	Affiliation	Work Experience	Responsibility	Type of Tasks (Assigned by the Study Team)
P1	40–49	female	Office for Integration and Migration	1–3 years	3–29 accommodation facilities	organisational
P2	40–49	female	NGO	1–3 years	n.a.	operational
P3	30–39	female	Office for Integration and Migration	4–10 years	3–29 accommodation facilities	organisational
P4	30–39	male	City administration	4–10 years	100–400 ASR	operational
P5	30–39	male	Office for Integration and Migration	1–3 years	3–29 accommodation facilities	organisational
P6	40–49	female	NGO	4–10 years	100–400 ASR	operational
P7	30–39	female	Office for Integration and Migration	1–3 years	>4000 ASR	organisational
P8	40–49	female	Office for Integration and Migration	4–10 years	>4000 ASR	operational
P9	30–39	female	Office for Integration and Migration	4–10 years	>4000 ASR	operational
P10	30–39	female	NGO	1–3 years	>4000 ASR	operational
P11	50–59	male	Health department	4–10 years	n.a.	organisational
P12	40–49	male	Office for Integration and Migration	4–10 years	100–400 ASR	organisational
P13	60–69	male	Health department	11–30 years	n.a.	organisational
P14	60–69	female	Health department	11–30 years	3–29 accommodation facilities	organisational

**Table 2:** Requirements for data dashboards expressed by the participants and their practical implications.

Requirements Mentioned for Data Presentation	Practical Implications	Present in the RESPOND-INTENT Dashboard (Yes/No/Can Be Improved)
***Content-related requirements***		
**Introductory text** *“Also, [refers to an introductory text] maybe just very briefly: What can I actually do with this dashboard, what functions are there, what can I find out with it? (…) [like] a short guide.”* (P10) *“[Information] on the origin (…), what the thought behind it is, what benefits it can have.”* (P4)	Dashboards should provide an introductory text with an explanatory and summarizing character so that users quickly understand what the data is about.	no
**Lay-person language** *“Exactly, so what I think would be very important for me is to make the dashboard accessible to people without scientific training. So, as far as possible, to try to use terms in the application of the dashboard (…) that one might use quite naturally.”* (P10)	Not all users might be familiar with technical terms (e.g., from the health field or statistics), thus the data display should use simple words so that lay persons may understand everything.	can be improved
**Relevant health indicators** *“I think it’s really good that this division into three relatively/or two very specific needs/that you have separated into physical and mental [health], that’s very important, because they are two completely different pairs of shoes. (…) That is extremely important. Otherwise it would really be a tool that would not be usable.”* (P5)	When selecting variables (e.g., health indicators) for a dashboard, their relevance to potential users should always be considered.	yes
**Comparative datasets** *“If you can then say “the [health] need here is much higher than the overall average”, that would also be something that helps with an argument.”* (P2) *“So, it is important, of course, to always look at what the relation to the general population is.”* (P13)	It is very helpful to include a comparative data set so that users can contextualise the data. This might be other population sub-groups or the general population.	no
***Technical requirements***		
**Interactive graphs** *“So, when I click on a district or a city and the statistics appear directly, I think it’s useful that I get the information directly.”* (P12)	Easily accessible pop-up windows are helpful to provide further information on the data graphs. In the case of RESPOND-INTENT it concerns the districts on the map, however this might also work for lines or bars in graphs.	yes
**Indicator information** *“And ideally, if I could wish, then in some way, when I move over here [the indicators and other terms]—such a beautiful bubble [window including more information] would somehow pop up.”* (P2)	Background information about important buzzwords/terms (e.g., indicators) should be easily accessible and easy to find. Ideally, pop-up windows could be used.	no
**Indicator selection and adjustment** *“So, it would be good if it could be possible to play around more with the data in order to get a little finer differentiation out of it.”* (P5)	Users might want to explore the given data in more detail, therefore functions for manual adjustments and possibilities for users to interact with the data would be recommendable.	can be improved
**Comparability functions** *“So, what we have just discussed, such a comparability between districts (…) is definitely not irrelevant.”* (P11)	Dashboards could include functions to compare data across different geographical regions. For example, in the case of RESPOND-INTENT this would be a function to compare values of different districts.	no
**Data export** *“So, what I would still like is if you could export them [the data], maybe as a pdf or also as an Excel file.”* (P10)	Users might want to use the information provided by the dashboards for other purposes. Thus, an export function as PDF, Excel file or graph would be recommendable.	no
**Timeliness and maintenance of data** *“The question would be, how is this maintained or how does the maintenance of this system work?”* (P4)	To contextualise the data provided by the dashboard, it is recommendable to provide information about the timeliness and maintenance of the underlying database.	no

## Data Availability

The data presented in this study are available on request from the corresponding author. The data are not publicly available due to data protection reasons.

## Supplementary Materials

The following supporting information can be downloaded at: https://www.mdpi.com/article/10.3390/ijerph19138049/s1, Supplementary File S1: Interview guide; Supplementary File S2: Code system; Supplementary File S3: Dashboard guide.
