# Supplementary material for: From Research into Practice: Converting Epidemiological Data into Relevant Information for Planning of Regional Health Services for Refugees in Germany
Source: Int J Environ Res Public Health. 2022 Jun 30;19(13):8049. doi: 10.3390/ijerph19138049 (PMC9265908; doi:10.3390/ijerph19138049)
Supplement: Supplementary file 1 [file ijerph-19-08049-s001.zip › Supplement_S1.pdf]

Supplementary File S1: Interview guide

Interview guide on healthcare planning for asylum seekers and refugees in Germany and the usability test for the RESPOND-INTENT dashboard

Part 1 – Data/Information basis for decision-making
**First of all, I would be interested to know your work position and what tasks your everyday work includes?**<br>• Which tasks relate directly to healthcare planning?<br>• How are you involved in the specific decision-making process?
Healthcare planning
**Can you describe how health care planning typically happens in your area of responsibility?**<br>• Who is involved?
**What challenges do you face in this respect?**<br>• How do you overcome those?
**Can you tell me about a situation where you yourself or in your county had to make a difficult decision?**<br>• Who was involved?<br>• Who made the decision? Who was ultimately responsible?
**What do you think works well about health care planning?**<br>• Where is a need for improvement?<br>• Can you give us an example where you have had good/bad experiences in planning?
Data/Information basis
Now you have already explained a lot about the planning process and the decision-making**. I would now be interested to know whether you use any data or information for this in your everyday work.**<br>• What kind of data do you use?<br>• And are there any that are health-related?<br>*If the answer is "No":*<br>• Are there reasons not to use data?<br>• On what basis do you make decisions?
**What data or information would you like to have in the future?**
**How would data or information need to be presented to support you in your planning or decision-making?**
Part 2 – Usability Test RESPOND-INTENT
We have developed a dashboard to estimate the regional burden of disease among refugees, called REPOND-INTENT or INTENT for short. The dashboard estimates the burden of disease in the counties of Baden-Württemberg, based on RESPOND data (2018). And we want to figure out how to make scientific data helpful and useful for decision makers. INTENT is an example of what this might look like. For the usability test that we will now go through, we will give you a scenario that you can use to try out the dashboard and its functions. We will ask you to share all your thoughts out loud, which may be unusual at first. But really speak out everything that normally only happens in your own head. This method is called "think aloud", so just think out loud. Since we are not recording a video and everything said will be anonymised later, it is important that you really say out loud everything you think, e.g. even if you are reading headlines or labels of buttons, just read that out. This helps us later to understand how it was for you, e.g. how difficult or easy it was to use INTENT and how comprehensible the dashboard is.<br><br>Do you have any questions?

**Before we will go through a scenario, first open the link to INTENT, the dashboard and describe what you see.**<br>• What you see now (in general): How does it appear to you?<br>• How does the colour scheme of the map appear to you?
Scenario
**You can now test the dashboard using the following scenario:**<br><br>Imagine you are planning health care for refugees in the Neckar-Odenwald district and are preparing for an important meeting with other stakeholders. You first want to find out what indicators the dashboard has and how they were actually formed. Look for this information. While doing this, don't forget to say all your thoughts out loud, including what you read, so we can follow along.<br><br>After you know more about the indicators, you are particularly interested in the psychosocial needs. Could you please compare the values of your district (Neckar-Odenwald district) with the values of Baden-Württemberg and a neighbouring district?
Further questions about usability, usefulness and data presentation
How easy or difficult is it to understand the information INTENT provides?
How likely would such a scenario, or one similar to it, be in your everyday work?
If you now think about your care planning: to what extent could INTENT support you in this?
Assuming we were to implement the dashboard in Baden-Württemberg, how would we need to inform about it?
We asked in the first part of the interview how data would need to be prepared to help you plan. Now you've learned about one way to present data, is there anything else you might have thought of?
