# Supplementary material for: From Research into Practice: Converting Epidemiological Data into Relevant Information for Planning of Regional Health Services for Refugees in Germany
Source: Int J Environ Res Public Health. 2022 Jun 30;19(13):8049. doi: 10.3390/ijerph19138049 (PMC9265908; doi:10.3390/ijerph19138049)
Supplement: Supplementary file 1 [file ijerph-19-08049-s001.zip › Supplement_S2.pdf]

Supplementary File S2: Code System

List of Codes	Frequency
Code system	716
Context	0
Description of organisation	15
Description of tasks	27
Healthcare planning	0
Actors’ tasks in healthcare planning	22
Description of collaboration in healthcare planning	29
Responsibilities in healthcare planning	6
Description of planning processes	10
Short-term planning	5
Long-term planning	11
Challenges in healthcare planning	37
Other	15
Decision-making basis	0
What kind of decisions are made?	4
Long-term decisions	19
Short-term decisions	11
Who decides?	16
Information basis for decisions	40

Desirable information	23
Other	9
Data	0
Communication and use of data	16
Requirements for data processing	11
Challenges in data preparation / retrieval	39
Political sensitivity of data	9
Data literacy (meta-level)	21
Other	13
Dashboard: technical details	0
Comprehensibility of the dashboard	0
Background information on the dashboard	24
difficult-to-understand terms	11
Transparency of data	22
Other	13
Functionalities	0
available	22
missing	25
Structure of the dashboard	40
Colour scheme	13
Technical problems	16
Other	9

Dashboard: content related issues	0
(Potential) target group (users)	6
Fields of use/purpose in everyday work	24
Desirable additional databases	0
(Other) vulnerable groups	2
General population	6
Specific pathologies	6
Children’s data	1
Other	22
How and who should be informed about the dashboard?	14
Other	9
Other	11
Usability test	10
