# Supplementary material for: From Research into Practice: Converting Epidemiological Data into Relevant Information for Planning of Regional Health Services for Refugees in Germany
Source: Int J Environ Res Public Health. 2022 Jun 30;19(13):8049. doi: 10.3390/ijerph19138049 (PMC9265908; doi:10.3390/ijerph19138049)
Supplement: Supplementary file 1 [file ijerph-19-08049-s001.zip › Supplement_S3.pdf]

Supplementary File S3: Dashboard guide

Dashboard to estimate regional health burden of asylum seekers in Baden-Wuerttemberg (RESPOND-INTENT)

Maren Hintermeier, Stella Erdmann, Kayvan Bozorgmehr, Louise Biddle| Heidelberg University Hospital

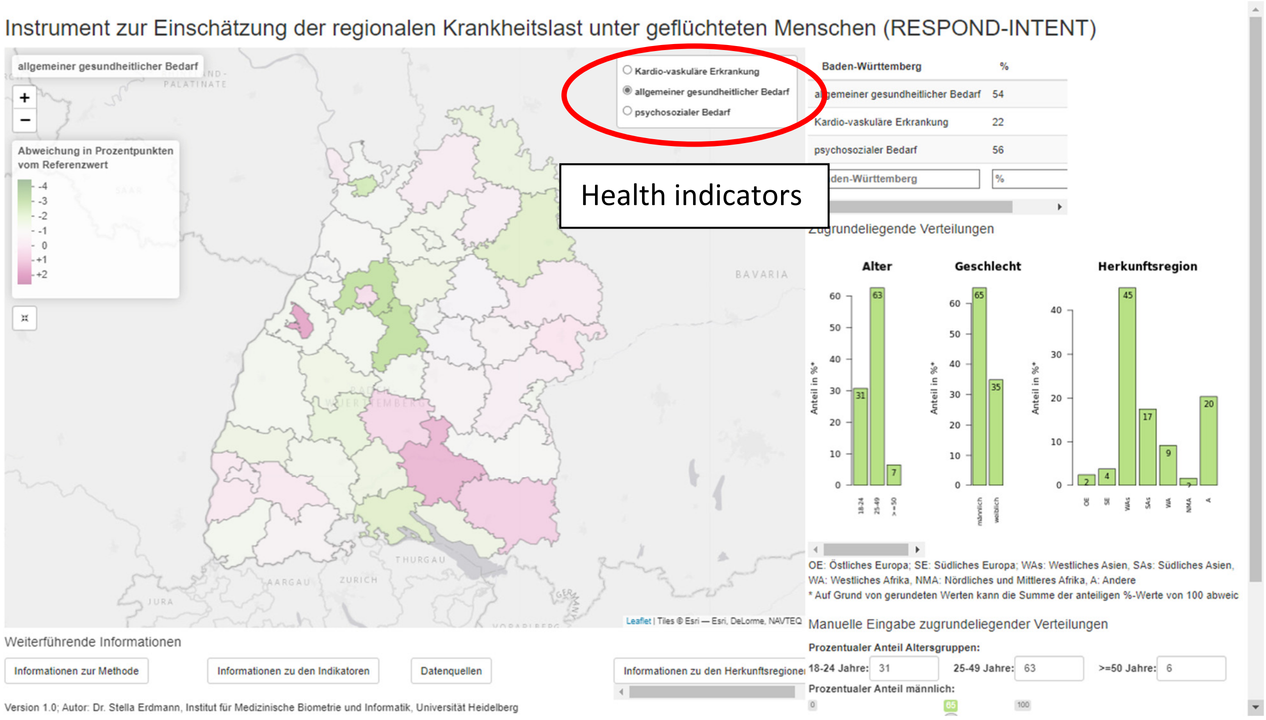

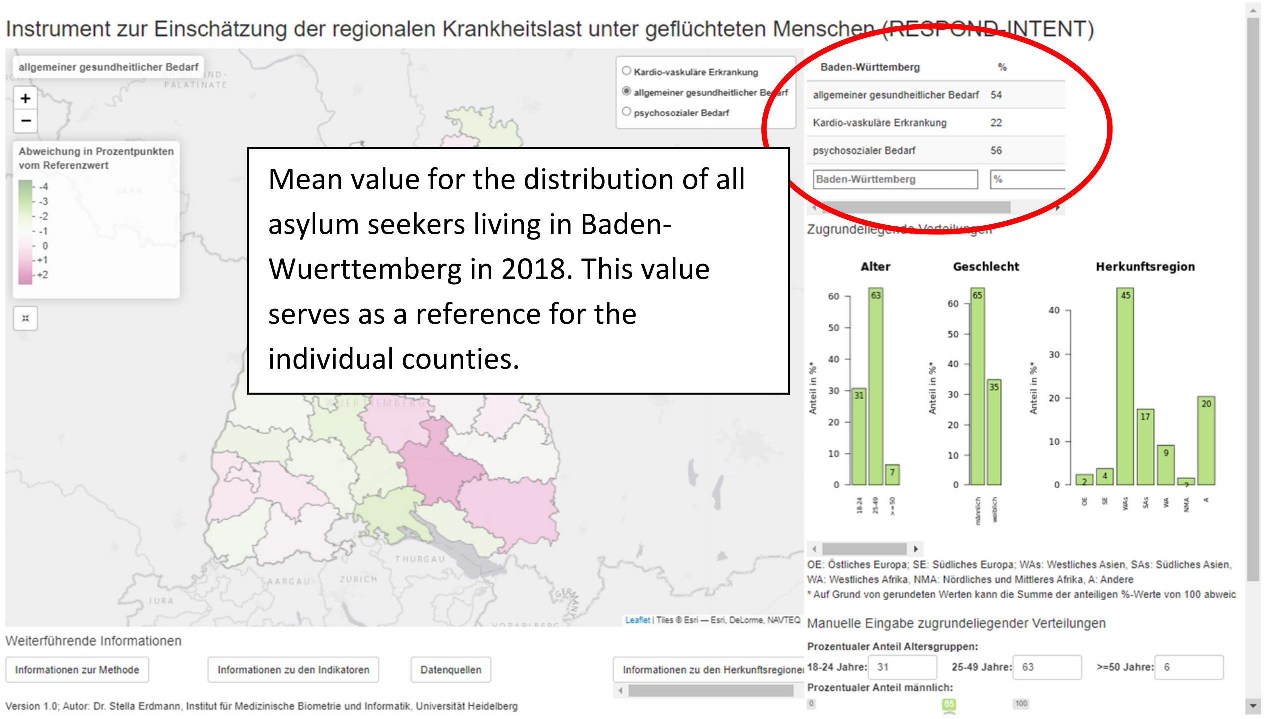

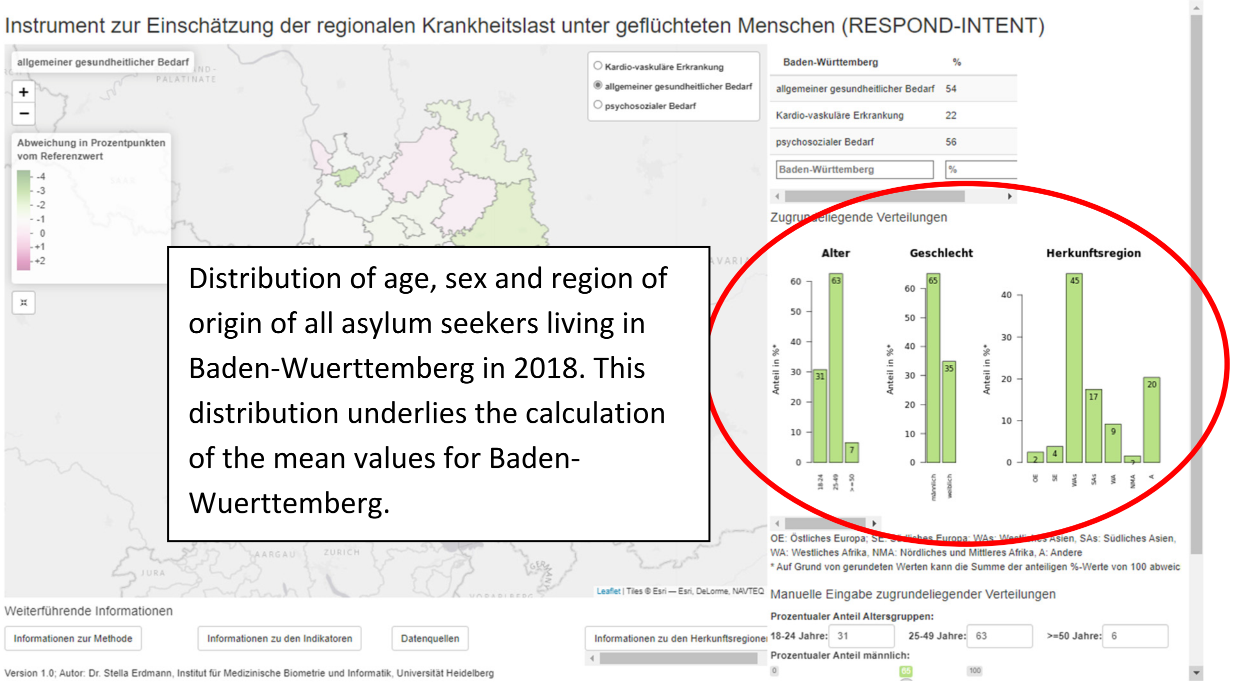
Instrument zur Einschätzung der regionalen Krankheitslast unter geflüchteten Menschen (RESPOND-INTENT)
allgemeiner gesundheitlicher Bedarf
Abweichung in Prozentpunkten vom Referenzwert
Kardio-vaskuläre Erkrankung
allgemeiner gesundheitlicher Bedarf
psychosozialer Bedarf
Baden-Württemberg %
allgemeiner gesundheitlicher Bedarf 54
Kardio-vaskuläre Erkrankung 22
psychosozialer Bedarf 56
Zugrundeliegende Verteilungen
Alter
Geschlecht
Herkunftsregion
Distribution of age, sex and region of origin of all asylum seekers living in Baden-Wuerttemberg in 2018. This distribution underlies the calculation of the mean values for Baden-Wuerttemberg.
Weiterführende Informationen
Informationen zur Methode
Informationen zu den Indikatoren
Datenquellen
Informationen zu den Herkunftsregionen
Manuelle Eingabe zugrundeliegender Verteilungen
Prozentualer Anteil Altersgruppen:
18-24 Jahre: 31
25-49 Jahre: 63
>=50 Jahre: 6
Prozentualer Anteil männlich:
Version 1.0; Autor: Dr. Stella Erdmann, Institut für Medizinische Biometrie und Informatik, Universität Heidelberg

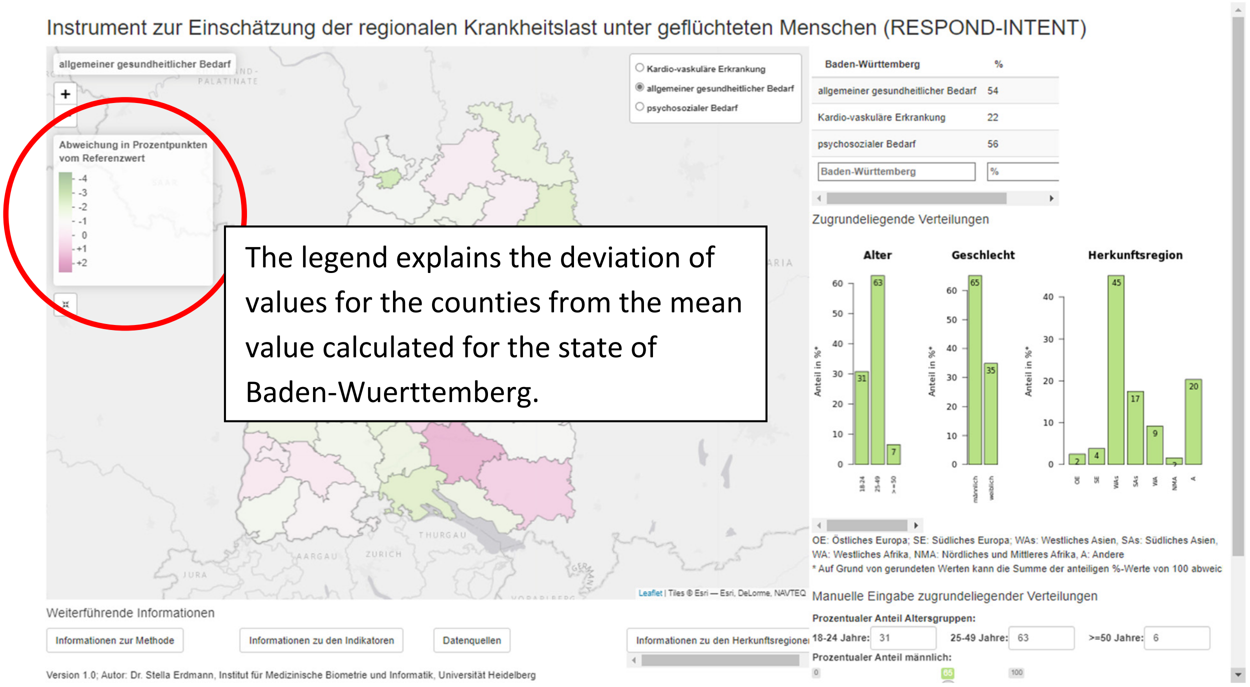
Instrument zur Einschätzung der regionalen Krankheitslast unter geflüchteten Menschen (RESPOND-INTENT)
allgemeiner gesundheitlicher Bedarf
Abweichung in Prozentpunkten vom Referenzwert
Kardio-vaskuläre Erkrankung
allgemeiner gesundheitlicher Bedarf
psychosozialer Bedarf
Baden-Württemberg %
allgemeiner gesundheitlicher Bedarf 54
Kardio-vaskuläre Erkrankung 22
psychosozialer Bedarf 56
Zugrundeliegende Verteilungen
Alter
Geschlecht
Herkunftsregion
The legend explains the deviation of values for the counties from the mean value calculated for the state of Baden-Wuerttemberg.
OE: Östliches Europa; SE: Südliches Europa; WAs: Westliches Asien, SAs: Südliches Asien, WA: Westliches Afrika, NMA: Nördliches und Mittleres Afrika, A: Andere
Weiterführende Informationen
Informationen zur Methode
Informationen zu den Indikatoren
Datenquellen
Informationen zu den Herkunftsregionen
Manuelle Eingabe zugrundeliegender Verteilungen
Prozentualer Anteil Altersgruppen:
18-24 Jahre: 31
25-49 Jahre: 63
>=50 Jahre: 6
Prozentualer Anteil männlich:
Version 1.0; Autor: Dr. Stella Erdmann, Institut für Medizinische Biometrie und Informatik, Universität Heidelberg

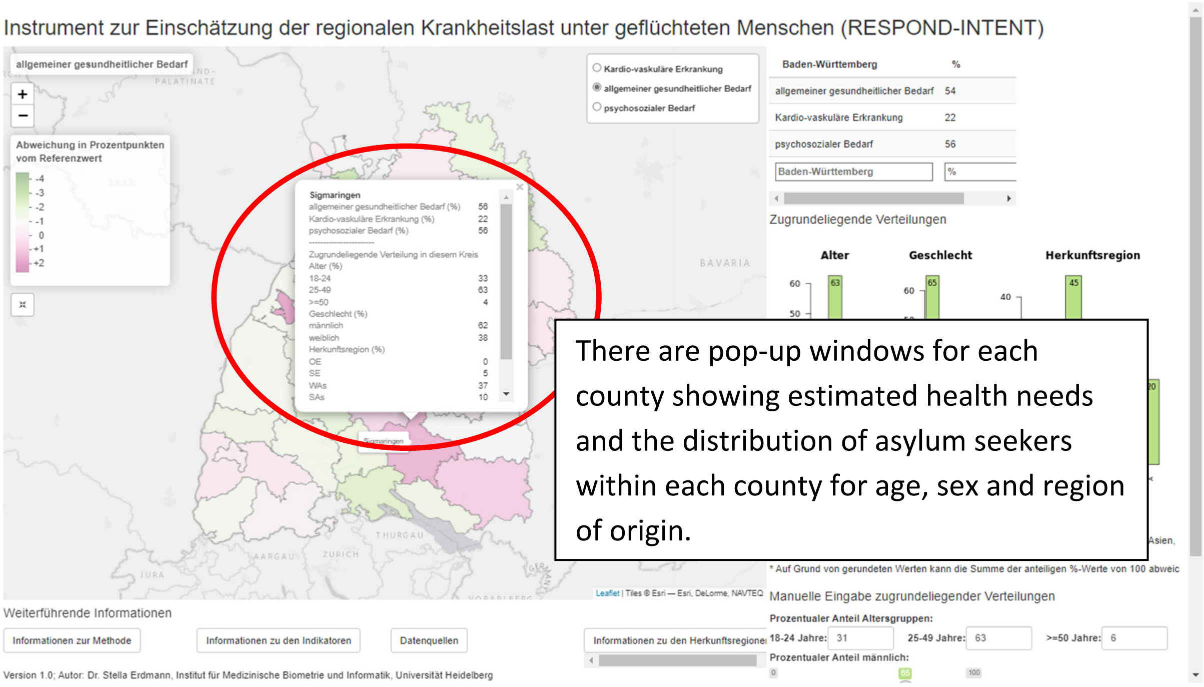

There are pop-up windows for each county showing estimated health needs and the distribution of asylum seekers within each county for age, sex and region of origin.

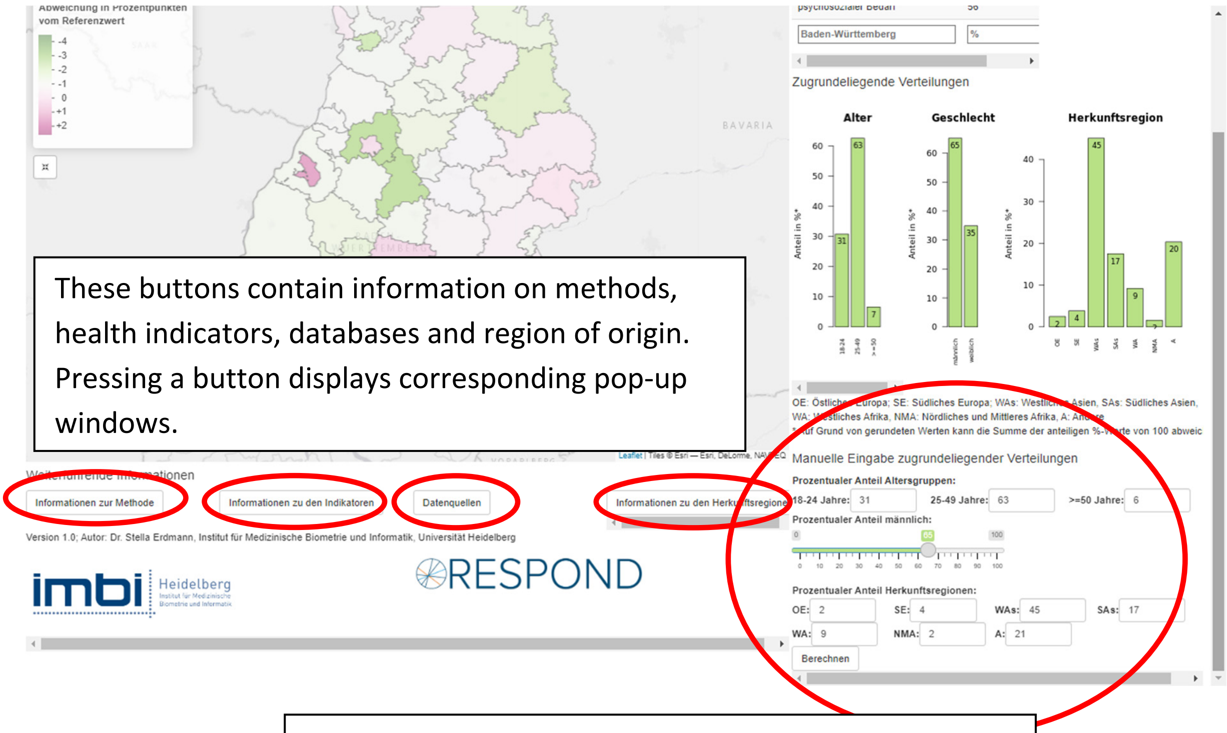

These buttons contain information on methods, health indicators, databases and region of origin. Pressing a button displays corresponding pop-up windows.

This is a field for manual entry. Since the underlying distributions are from 2018, they may have changed. Therefore, we offer this manual entry feature to enter the current distributions for age, sex, and region of origin. The values are then calculated accordingly and displayed at the top of the page.
